# Economic assessment of US physician participation in short-term medical missions

**DOI:** 10.1186/s12992-016-0183-7

**Published:** 2016-08-22

**Authors:** Paul H. Caldron, Ann Impens, Milena Pavlova, Wim Groot

**Affiliations:** 1 Maastricht Graduate School of Governance, University of Maastricht, Maastricht, Netherlands; 2 Midwestern University, Institute for Healthcare Innovation, 555 31st Street, Downers Grove, IL 60515 USA; 3 Department of Health Services Research, CAPHRI, Maastricht University Medical Center, Faculty of Health, Medicine and Life Sciences, Room 0.073, Duboisdomein 30, 6229 GT Maastricht, Netherlands; 4 Department of Health Services Research, CAPHRI, Maastricht University Medical Center, Faculty of Health, Medicine and Life Sciences, Room 0.033, Duboisdomein 30, 6229 GT Maastricht, Netherlands

**Keywords:** Medical missions, Short-term, Transnational aid

## Abstract

**Background:**

Short term medical missions (STMMs) are a form of unregulated and unsanctioned, grass roots, direct medical service aid from wealthier countries to low and middle income countries. The US leads the world in STMM activity. The magnitude of monetary and man power inputs towards STMMs is not clear. The objective of this study is to estimate the prevalence of physician participation in STMMs from the US and the related expenditures of cash and resources.

**Methods:**

An online survey solicited information on physician participation in STMMs. Responses regarding costs were aggregated to estimate individual and global expenditures.

**Results:**

Sample statistics from 601 respondent physicians indicate an increasing participation by US physicians in STMMs. Including opportunity cost, average total economic inputs for an individual physician pursuing an STMM exceed $11,000. Composite expenditures for STMM deployment from the US are estimated at near $3.7 billion annually and the resource investment equates with nearly 5800 physician fulltime equivalents.

**Conclusions:**

STMM participation and mission numbers have been increasing in the millennium. The aggregate costs are material when benchmarked against formal US aid transfers. Understanding the drivers of physician volunteerism in this activity is thereby worthy of study and relevant to future policy deliberation.

## Introduction

Short-term medical mission (STMM) refers to an activity wherein physicians and other medical workers from higher income countries travel to provide direct care to persons in lower and middle income countries (LMICs)^1^ without compensation for a period of days to a few weeks. Such “missions” are planned, and represent an unsanctioned, grass-roots, and highly direct expression of transnational aid. These medical excursions are differentiated from ad hoc responses to domestic or external disasters, full-time relief practice such as Médecins Sans Frontières (MSF), military or other governmental relief expeditions and officially sanctioned medical student or residency training programs.

In their systematic review in 2012, Martiniuk et al identified the USA as the most prolific among the four leading sending countries for STMMs that include Canada, United Kingdom and Australia [1]. Further, published articles suggest that overall STMM activity is increasing, though evidence for such an increase is not ultimately identified in the literature [1–3].

In the absence of available secondary sources of data specific to physician participation in STMMs, our Physicians’ Giving Back Survey (PGBS) was designed to assess an array of US physician volunteerism [4]. Embedded in the survey were questions related to physician participation in STMMs including demographic characteristics and costs associated with participation. The objectives of this study were to quantify participation in and evaluate the economic dimensions of STMMs from the US through analysis of data provided by respondents to the PGBS. Quantifying incident STMM participation and respondents’ itemized direct costs allows for the objective estimation of global expenditures related to STMMs. The effects of opportunity cost are also included in the calculation. Shaping an estimate of the global costs of US physician participation in STMMs by sensitivity analysis allows reflection on the relative materiality, i.e., the composite size of the monetary costs and manpower investment, of this form of aid.

Corroborating the impression that STMM activity sent from the US is increasing is important for several reasons. Rich country physicians are generally welcomed to provide services in LMICs under the guise of intercultural exchange. Usually physicians must provide documentation of current, active licensure in the sending country via a host organization to health authorities, but do not become licensed in the host country. While the intention is primarily to provide free direct medical and surgical care, preventative health services, and both professional and patient education for the benefit of the ostensibly poor, the potential harms in receiving communities have been well characterized [1]. These harms may include, among others, inadequate follow-up care, inadequate informed consent, therapeutic misadventures when visiting physicians act beyond their training or usual scope of practice, competition with or displacement of local providers of care, and dependency of communities and health authorities on traveling teams that inadvertently dampens local government investment in health resources. These risks must be outweighed by demonstrable benefits, but this assessment is stymied by the reality that the beneficial impact to community health and economics resulting from STMMs have not been measured, and are perhaps not always measurable [3]. Since regulatory oversight of these trips on both sides is generally weak, the composite risk may rise while the methodologies and case models to assess benefit to communities remain immature. Further, if this civil society effort to advance healthcare in LMICs through STMMs were found to be relatively effective in comparison to direct foreign aid, it may be prudent for rich countries, including the US, to refine and exploit this outreach for the benefit of global health, as well as for the potential projection of diplomatic soft power, i.e., the capability of attracting and winning the favor of communities by means other than coercion [5]. Lastly, with a rise in the number of STMM and physician participants could come a greater potential for adverse incidents to do harm, and to come to public attention in an unfavorable light before the positive dimensions of STMMs are fully appreciated.

As with data on prevalence of physician participation, composite secondary sources of data to accurately assess overall costs are also not readily available. In the evaluation of the return on investment in STMMs, provider side cost data remains easier to collect than receiver side health or economic impact measurements because of the fragmented nature of STMM activity in its present state. This study is not intended as a comprehensive review or adjudication of all aspects of STMMs, nor is it powered to provide both sides of a risk-benefit equation. The importance of the data from our survey is to provide useful information for the input side of such an analysis through the methodology of direct solicitation from participating physicians, and to provide researchers, policy makers, and physicians a sense of the magnitude of these inputs.

## Background

Prevalence and incidence data on STMM participation from the US is sparse. Personal communications (December 2013) with administrations of the American Medical Association (AMA), the American College of Physicians and the American College of Surgeons for information from their respective demographic data bases, reveal that no attempt has been made to gather data on STMM participation of their memberships.^2^ The activity is not sanctioned by any common social organization or regulatory agency. While the expenditures are generally tax exempt in the US, there are no identifiers within the US federal tax code that specifically mark these outlays in a manner that would allow them to be tabulated nor to project a demographic profile of persons who claim exemptions specifically related to STMM activity.

A 2012 survey Medscape of WebMD revealed that approximately 10 % of physicians participate in some form of “international mission work” although STMMs were not distinguished from other formats such as long term medical work, evangelism, habitat construction or other activities [6]. In a similar Medscape survey question in 2014, the overall rate of unspecified international volunteer activities were nearer 7.5 % [7].

Due to the absence of sources of pooled data from individuals, governmental agencies, non-governmental organizations (NGOs) or medical professional societies, the economic resources expended for STMMs from the US are not readily calculable. Physicians volunteering for STMMs are generally expected to cover their own personal expenses and pay organizational fees to support administrative and other costs. Personal costs categorically include airfare, the cost of supplies or equipment that the physician may personally utilize or expend in the course of providing direct patient services, as well as organizational fees that apply to administrative costs, tolls, duties, other in-country facilitation fees, billeting and ground transportation costs. Further, the majority of participant physicians are gainfully engaged in practices where the opportunity cost, i.e., the amount of income foregone during their absence for a period of time, can be estimated. Herein we report findings from the PGBS sample on estimated participation rate and itemized and global costs. Potential policy implications of the results are discussed.

## Methods

Survey methodology for the PGBS has been previously described [4]. The PGBS was conducted as an online survey in 2014 in the US. Beta testing of the PGBS was performed utilizing a selected group of 15 identified physicians whose critiques were incorporated into the final PGBS version. Exempt status was granted for use of human subjects for the survey from the Investigational Review Board of Midwestern University Office of Research and Sponsored Programs, Downers Grove, Illinois, USA. The survey was implemented through SurveyMonkey©. Deployment of the survey to 109,237 unique physician emails was executed between January 30 and February 27, 2014. Response reception was closed on 30 April 2014. The email list included only physicians who were licensed to conduct the full spectrum of medicine (US MD, IMG, and DO).^3^ The survey targeted 93 % MDs/IMGs and 7 % DOs, proportionate to the US physician population distribution as provided in the American Medical Association 2011 Physician Master File (data as of Dec. 31, 2010). The survey was disseminated equally to the four regions of the US.^4^ Sample physicians were further targeted by specialty in close proportion to the specialties represented in the US physician population [8]. The proprietary email database of Healthcare Data Solutions (HDS) was used. The HDS database conforms to industry best practice guidelines for business-to-business email acquisition, adheres to US CAN-SPAM guidelines and maintains a quarterly “permissioning” and validation process. HDS’s DirectSelect tool herein eliminated titles such as Doctor of Chiropractic, Doctor of Optometry, Doctor of Podiatric Medicine, Licensed Acupuncturist, Naturopathic Doctor, dentists and PhDs.

The first question of the survey screened for a target sample of physicians that had completed all formal training and are or had been in practice in the US, followed by questions to capture participation in a variety of activities related to physician skills. Respondents who affirmed STMM participation were then directed to alternative pathways dependent upon single-mission versus multiple-mission participation. Using a series of ranges, physicians in the single-mission group were asked to provide numerical information on income at the time of the mission, airfare expenditures and organizational fees paid, equipment costs and opportunity costs. Multiple- mission participants provided these amounts for their first and most recent missions. In order to avoid discouraging completion of the lengthy survey, the PGBS asked physicians to provide accurate monetary information to the best of their ability without extensive review of personal records. Opportunity costs refer to a scaled estimate by the physician of the amount of revenue not generated in his or her practice as a result of the time away on the STMM. Means were calculated based on the weighted midpoints of each US dollar amount range; for the top ranges using a dollar figure “and above”, the bottom value to the range was used as the multiplier.

The Statistical Package for the Social Sciences (SPSS) version 22 was utilized in the chi square test comparisons of sample and population characteristics. The social studies proprietary statistical software STATA version 12 and Excel® proprietary software were utilized throughout the analysis of data.

## Results

In total, 631 filled-in questionnaires were received of which 601 fit the target criteria and could be used in the study. The sample and the physician population of the US were statistically similar with respect to race, civil status, and type of medical degree (*p* < 0.05, 95 % CI), dissimilar with respect to gender, age, whether trained in the US or abroad, religion, and region of the country (chi square test), and comparable in rank order with respect to the top 16 of 29 specialties in the population [4]. At the time of the survey in the first quarter of 2014, 32 % of the physicians in our sample indicated that they had taken part in one or more STMMs. Table 1 shows the prevalence of participation in the PGBS sample within decreasing retrospective periods through 2013, the final year of complete data from the PGBS. Also displayed in this table is the participation rate for 2012, the latest year for which state-based physician population data needed for subsequent calculations is available. The table suggests that the occurrence of new participation is intensifying.It should be noted that the physician participation rates are based on the total STMMs executed in each year and assumes one mission per year per physician; it is likely that a small number of physicians may participate in more than one STMM in a year which would lower the derived annual percent participation. On the other hand, respondents were asked to list the years of their first 10 STMMs only. Thirty-four of 170 STMM participants had been on more than 10, meaning that 417 STMMs without specified years were thereby not included in the representation figures (757 total mission by these 34 physicians; 757 – (34 x 10) = 417) with the possible effect of deflating annual participation rates in some years. It is also noteworthy that STMMs deployed to Haiti in 2010-2013 showed a 49 % rise (61/41) over the 1967-2009 period following that country’s major earthquake.

**Table 1:** Mean annual percent STMM participation over defined periods

Period	Period Mean Annual % physician participation in STMMs	Retrospective period
1973-2013	5.7 %	40 years
1983-2013	6.8 %	30 years
1993-2013	8.7 %	20 years
2003-2013*	12.5 %	10 years
2012**	16.5 %	2012 only

*Latest year for which complete PGBS data collected

**Latest year for which state-based physician population data available

**Fig. 1:**
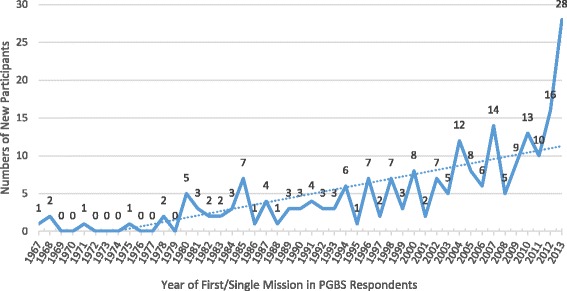
Trend in New Physician STMM Participation

**Fig. 2:**
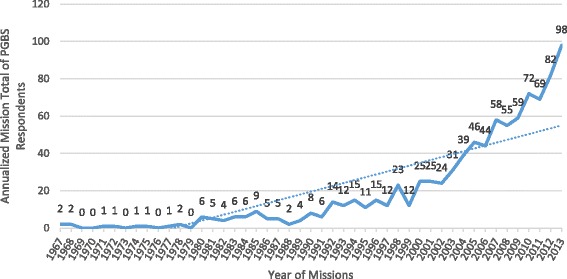
Trend in Annual Total Missions by Physicians

**Fig. 3:**
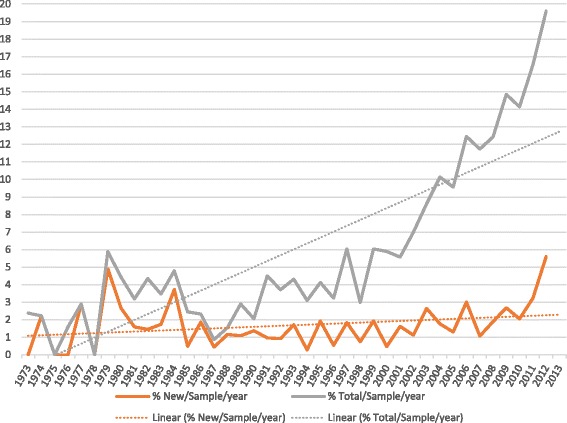
Percent of New and Total Mission Participants per Total Respondents in sample by Year

Figures 1 and 2 respectively illustrate the increasing prevalence of new first-time STMM participation as well as overall increasing numbers of missions by PGBS respondent physicians during the period 1967 – 2013. Figure 3 tracks new mission participants as well as the total mission participants as a percent of the survey respondents in practice at each year. The orange trend line illustrates that the percent of sample physicians that participate in missions for the first time is keeping up with, if not slightly exceeding, the number of physicians entering practice during each survey year. Further, the gray trend line illustrates that the total number of PGBS respondents participating in a mission each year is increasing relative to the respondents in practice for the survey year. The results are in range with the Medscape 2012 Physician Lifestyle Survey that suggested a roughly 10 % incidence in international mission work by US physicians in recent years.

Mission length for PGBS respondents averaged 11.8 days (range 1–90 days, SD 10.3 days) for the 908 of 926 STMMs wherein the duration was reported.

**Fig. 4:**
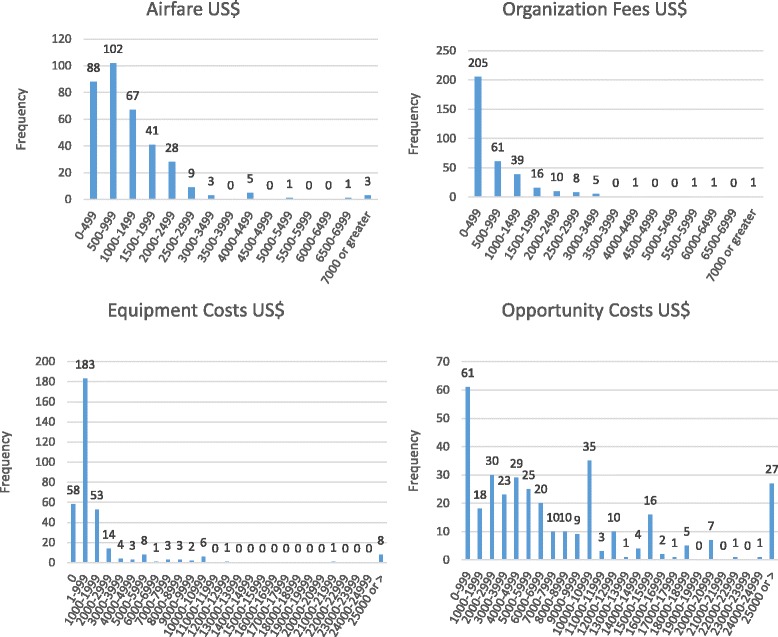
Distributions of itemized costs

Single-mission PGBS participants provided monetary information on 49 missions and multiple- mission respondents provided cost information for an additional 299 missions. Table 2 displays average airfare expenditures ($1165.45), organizational fees ($787.58) and equipment costs ($1861.93) reported by PGBS respondents. Average individual opportunity cost in the PGBS was $7791.21. Figure 4 illustrates the distribution characteristics for these costs. Using these calculated means, direct expenditures for a typical STMM in US$ at the time of mission (not adjusted for inflation) were $3814.96. If one includes opportunity cost, total economic inputs for an individual physician pursuing an STMM would be $11,606.17.

**Table 2:** Itemized estimated STMM costs

Item	Mean	SD	Range
Airfare	$1165	+/− $1035	$249.50–7000.00
Organization fees	$740	+/− $881	$249.50–7000.00
Equipment costs	$1861	+/− $4256	$0–25,000
Opportunity costs	$7791	+/− $7173	$499.50–25,000

When asked about the tax ramifications of the personal direct expenses for STMM activity, 63 % indicated a belief that deductibility from personal income was less than 100 %.

### Sensitivity analysis

To assess the scope of outlays by US physicians that accrue to conduction of STMMs and to ponder their materiality, respondents’ direct and opportunity costs in years 1983, 1993, 2003 and 2012 were multiplied by the estimated STMM participation for those years. US physician participation was estimated by multiplying the percent sample participation and the US physician population recorded in the respective year. Table 3 provides the resultant calculations of total costs without and including opportunity cost. The totals trend upward from 1993 reaching a half billion US$ in direct expenditures by 2012 and over four times that amount when opportunity costs are included.

**Table 3:** Sensitivity analysis of global annual costs

Sample year	PGBS Obs.	Direct Costs	Opportunity Costs	Total	Participation in Sample year (%)	US Physician Population in Sample year	Estimated physician STMM participation	Global US physician costs	
								Direct (airfare, organizational fees, equipment)	Total (Direct and Opportunity costs)
1983	2	$1,999	$14,250	$16,249	4.3 %	542,000*	23,565	$47,100,978	$382,899,435
1993	3	$1,999	$5,166	$ 7,165	3.7 %	703,700**	26,063	$52,095,519	$186,741,130
2003	4	$2,124	$9,000	$ 11,123	7.0 %	871,500***	60,848	$129,218,277	$806,037,886
2012	19	$3,907	$10,368	$14,275	16.5 %	878,194****	145,185	$567,170,189	$2,072,451,362

*Statistical Abstracts of the United States 1986. p103

**Statistical Abstracts of the United States 1995. p121

***Statistical Abstracts of the United States 2006. p113

****Federation of State Medical Boards 2012

Sensitivity analysis is used to determine how different values of an independent variable (herein ancillary staff) may impact a particular dependent variable (herein total cost) under a given set of assumptions. Some physicians may carry out STMMs alone or in a small team of physicians, acquiring the assistance of local personnel where necessary to provide care [9]. It is more likely, however, that each physician embarking on a trip is accompanied by one or more ancillary personnel to assist with medical or administrative functions. The direct costs for the additional personnel may be covered by the physician or other means. Regardless of source of funding, the travel and organization costs for the ancillary persons may be assumed to be equal to those of the physician. Equipment costs and opportunity costs would not apply to ancillary personnel. In Table 4, we have re-enacted projected global costs adding in the outlays for 1–4 ancillary personnel per physician. This sensitivity analysis, via the multiplier effect of ancillary staff, witnesses the global cost of missions expanding as much as 50 % of all-inclusive costs for the physician alone, exceeding $3.7 billion.

**Table 4:** Cost estimates including ancillary personnel* with physician costs

Ancillary Personnel Calculation									
Sample year	Airfare	Organization fees	Total (direct)	Estim. participation					
				AncillaryX1	Ancillary direct	Physician direct	Physician+ A1 direct	Physician total	Physician + A1 Total
1983	$ 1,500	$ 250	$ 1,749	23565	$ 41,215,565	$ 47,100,978	$ 88,316,543	$ 382,899,435	$ 424,115,000
1993	$ 1,583	$ 250	$ 1,832	26063	$ 47,756,036	$ 52,095,519	$ 99,851,555	$ 186,741,130	$ 234,497,165
2003	$ 875	$ 875	$ 1,749	60848	$ 106,423,105	$ 129,218,277	$ 235,641,381	$ 676,819,609	$ 783,242,714
2012	$ 1,881	$ 947	$ 2,828	145185	$ 410,587,845	$ 485,076,783	$ 895,664,627	$ 2,072,451,362	$ 2,483,039,207
				AncillaryX2	Ancillary direct	Physician direct	Physician+ A2 direct	Physician total	Physician + A2 Total
1983	$ 1,500	$ 250	$ 1,749	47130	$ 82,431,130	$ 47,100,978	$ 129,532,109	$ 382,899,435	$ 465,330,565
1993	$ 1,583	$ 250	$ 1,832	52126	$ 95,512,072	$ 52,095,519	$ 147,607,591	$ 186,741,130	$ 282,253,201
2003	$ 875	$ 875	$ 1,749	121696	$ 212,846,209	$ 129,218,277	$ 342,064,486	$ 676,819,609	$ 889,665,819
2012	$ 1,881	$ 947	$ 2,828	290371	$ 821,175,689	$ 485,076,783	$ 1,306,252,472	$ 2,072,451,362	$ 2,893,627,052
				AncillaryX3	Ancillary direct	Physician direct	Physician+ A3 direct	Physician total	Physician + A3 Total
1983	$ 1,500	$ 250	$ 1,749	70696	$ 123,646,696	$ 47,100,978	$ 170,747,674	$ 382,899,435	$ 506,546,130
1993	$ 1,583	$ 250	$ 1,832	78189	$ 143,268,107	$ 52,095,519	$ 195,363,627	$ 186,741,130	$ 330,009,237
2003	$ 875	$ 875	$ 1,749	182544	$ 319,269,314	$ 129,218,277	$ 448,487,591	$ 676,819,609	$ 996,088,924
2012	$ 1,881	$ 947	$ 2,828	435556	$ 1,231,763,534	$ 485,076,783	$1,716,840,316	$ 2,072,451,362	$ 3,304,214,896
				AncillaryX4	Ancillary direct	Physician direct	Physician+ A4 direct	Physician total	Physician + A4 Total
1983	$ 1,500	$ 250	$ 1,749	94261	$ 164,862,261	$ 47,100,978	$ 211,963,239	$ 382,899,435	$ 547,761,696
1993	$ 1,583	$ 250	$ 1,832	104252	$ 191,024,143	$ 52,095,519	$ 243,119,662	$ 186,741,130	$ 377,765,273
2003	$ 875	$ 875	$ 1,749	243392	$ 425,692,419	$ 129,218,277	$ 554,910,696	$ 676,819,609	$ 1,102,512,028
2012	$ 1,881	$ 947	$ 2,828	580741	$ 1,642,351,378	$ 485,076,783	$ 2,127,428,161	$ 2,072,451,362	$ 3,714,802,741

* “A1, A2, A3, A4” in the table connotes the accompaniment by 1–4 ancillary personnel

## Discussion

PGBS responses confirm that incident US STMM participation and mission numbers may have been increasing in the new millennium consonant with the assertions of previous reports [1–3].

Most estimates of STMM cost found in current literature relate to single missions, single persons, single NGOs or the care of single patients rather than tackling global expenditures [9–12]. Maki et al estimated an annual global cost of at least $250M expended on STMMs from the US based upon an internet search to estimate the number of mission organizations, an average team cost per mission and average number of missions per organization per year. While far less reliable than invoice data, our sensitivity analysis based on primary costs and opportunity costs reported by sample respondents provides a more credible estimate of direct and global costs. The calculations indicate cost figures that are magnitudes greater than previously estimated. Our data set includes only that from respondent physicians qualified by our survey; the inclusion of the multiplier of ancillary personnel draws a more realistic picture of global costs, currently exceeding US$3.7 billion. A perspective on such a sum is facilitated by other aid benchmarks from United States Agency for International Development (USAID): total US aid to Afghanistan ($3.1B), contributions to International Organizations and Peacekeeping Activities ($3.7B), Consular Affairs and the Border Security Program ($2.8 B), Humanitarian Assistance ($4.1B) and the Global Health Initiative ($8.3B) [13]. Of course, these USAID disbursements are actual costs, funded through the US tax base, and do not involve opportunity costs as do our estimates for global STMMs expenses. On the other hand, the missed opportunities for all of these expenditures may be found in much needed US domestic infrastructure renovation, educational and research investments.

Nearly 63 % of STMM participants in our sample believed that their expenses were only partially deductible from earned income, while in reality such expenses are generally fully deductible from taxable income. The deductibility of the costs creates a de facto federal and state subsidy for STMM activity which remains free from regulation, sanction or outcome measures. In some cases, academic physicians may be subsidized by their institutions if there can be demonstrated a teaching component to their STMM activity, thus resulting in less or no out of pocket costs to the physician, and such salaried physicians may be immune personally to opportunity costs.

Mission length for PGBS respondents averaged 11.8 days (range 1–90 days, SD 10.3 days) for the 908 of 926 missions reported. Extrapolating the 16.5 % of responders who participated in missions in 2012 to the population of physicians at that time, each working for 10 days (of an 11.8-day average mission span), the investment of physician work days expended then approaches 1,451,850 working days or 5784 physician full time equivalents from the US physician work force [(145,185 X 10 = 1,451,850 days)/251 work days in a year]. In a country that faces a chronic manpower shortfall of tens of thousands of physicians, such a transfer of resources may be meaningful [14].

Our evidence suggest that STMMs are on the rise with considerable composite and personal economic and manpower inputs. Could this arc herald an underutilized dimension for advancing global health in front of direct foreign aid and relief in the face of disaster or conflict? If so, then the fragmentation of the current effort will undoubtedly require a transformation wherein the STMM dimension adopts the disciplined allocation of resources of long-term humanitarian groups like MSF, and the registration and vetting of participants that the World Health Organization (WHO) has begun in order that countries facing disaster can know that the relief is coming from reliable sources [15]. A national or international organization focused specifically on STMMs (non-emergent), as defined in our Introduction, could follow the WHO’s emergency work force lead by registering and vetting willing private practice physicians as a basis for facilitating the match of skills to need in LMICs and integrating guidelines, quality assessments, and data collection protocols not only for surgical, but also for medical STMMs.

Several groups have advanced the arduous work of the differentiating quality among surgical relief trips, and a body of knowledge is accumulating on the “how to” of execution of STMMs and the importance of long term collaborative efforts [16–22]. These reports mostly address comparative effectiveness of individual trip models, patient selection for procedures, informed consent, and tracking surgical outcomes. While beyond the scope of this study, what must follow is the development of methods to assess the cumulative effects on individual LMICs that all repeating STMM sources may have, and that will require intensifying social and public health research on this subject.

The increase in STMM activity and the related expenditures has several policy implications. If deductions for charitable activities are eliminated from the tax code, this could reduce the incentive for some physicians to start or continue participation. Government could ultimately determine that the cross-border ramifications of these care transfers, both in terms of lost tax revenue and the diversion of economic output and healthcare resources from the US, are substantive, and begin a process of registry and/or regulation. These should be considered as contingent scenarios that are unlikely in the near term. More realistic may be the fostering of this humanitarian activity for soft-power purposes among its neighbors and non-neighbors, even though the physicians themselves may have no diplomatic agenda. Since improving health status promotes economic status, STMMs, if deemed effective, could eventually be viewed among the policy tools of wealthy countries to support development in LMICs [23]. Registration and credentialing of participants, matching of the skillsets of volunteers to the areas of need, whether through government or a professional association dedicated specifically to STMMs, may be the steps that reduce fragmentation of effort among STMM participants and NGOs, optimize the return on the growing investment, and promote its sustainability. Narrative research on the positive views of recipient communities are accumulating [2, 24–26]. If future assessments of the impact on health and economic status of communities are ultimately positive, then understanding the personal drivers of physician involvement may aid in recruitment strategies as well as matching skills to community needs. Last, but certainly not least, a benchmark of the personal costs, along with a broader current perspective on STMMs, may help physicians in their own deliberation over involvement. An appreciation of the ramifications of STMMs, not only to individual patients, but also to the context of global health, may inform physicians’ cooperation in organizations that act on this broader perspective.

The assessments of our survey data should be looked upon with caution since such surveys are inherently susceptible to response bias, respondent recollection and reporting bias. Response bias, along with the assumption of one STMM per physician per year, may inflate both the total reported STMM participation (32 %) and the annual percent physician participation of the population, whereas the sizable number of STMMs for which the year was not reported and thereby excluded from the assessment may deflate our calculated rate of participation. While the trend over the prior 40 years to 2013 affirms increasing participation in STMMs by US physicians, a bump in trips to Haiti in the four years following its 2010 earthquake may inflate the later numbers, though the traveling physicians may as readily have gone elsewhere were it not for that disaster. The respondent sample significantly correlated with US physician with respect to only 3 of 8 demographic and professional characteristics. In addition, monetary answers were solicited through selection of ranges. Therefore, individual and composite amounts are weighted estimates. We utilized ranges of dollar amounts provided by respondents and made no attempt to actualize for time value of the dollar over the reporting period. Further caution is warranted because of the small sample size and recall bias on the part of respondents. While response bias may inflate the composite cost estimates to some degree, the failure to capture some costs, such as the sizable expense of multiple travel vaccinations, malarial prophylaxis, grants and donations-in-kind by pharmaceutical companies, surgical devices or disposables given by manufacturers, hospitals, and charities, and other uncaptured expenditures by some participants may lead to underestimation. Some ancillary personnel may incur opportunity costs from unpaid time off work; no attempt was made herein to assess or include that potential sum.

## Conclusions

The rates of STMM participation found in the PGBS data support prior speculation in the literature that incidence and prevalence of physician participation and mission numbers have increased. Utilizing our physician reported cost data, the annual composite outlays for US STMMs comprise a material investment in this form of unofficial aid, both economic and related to allocation of relatively scarce manpower resources, while being indirectly subsidized through the taxation regime. Representing one of the most highly educated sectors of society, physicians are increasingly finding the trade-offs worthwhile in the non-remunerative exchange of STMMs. The materiality in terms of monetary cost and manpower justifies the effort to understand physician motivation to participate in STMMs, and merits monitoring going forward, and may influence policy considerations intended to optimize cost benefit and non-economic utility of STMMs.

## Abbreviations

AMA, American Medical Association; IMG, International Medical Graduate; NGO, non-governmental organization; PGBS, Physicians’ Giving Back Survey; STMM, short-term medical mission

## Footnotes

1 World Bank list of economies, February 2014, siteresources.worldbank.org/DATASTATISTICS/Resources/CLASS.XLS

2 AMA is the professional association that represents the general interests of licensed American physicians. ACP is the professional organization for American physicians with training in non-surgical and non-obstetrical medical care of adults. The ACS is a professional organization that represents the general interests of surgeons in the US.

3 “US MD” refers to physicians who received their Doctor of Medicine degree from a U.S. allopathic medical school. “DO” refers to physicians who received a Doctor of Osteopathy degree from a US-sanctioned osteopathic medical school. International medical graduate (IMG) refers to an individual with an MD or equivalent degree who graduated from a medical school outside the United States and its territories or Canada, including U.S. citizens who have attended such medical schools abroad. To be eligible for licensure and practice in the United States, all IMGs must have completed accredited graduate training in the United States.

4 The Northeast includes nine states: Connecticut, Maine, Massachusetts, New Hampshire, Rhode Island, Vermont, New Jersey, New York and Pennsylvania; the Midwest region includes twelve states: Illinois, Indiana, Iowa, Kansas, Michigan, Minnesota, Missouri, Nebraska, North Dakota, Ohio, South Dakota and Wisconsin; the Southern region includes sixteen states: Florida, Georgia, Maryland, North Carolina, South Carolina, Virginia, West Virginia, Delaware, Alabama, Kentucky, Mississippi, Tennessee, Arkansas, Louisiana, Oklahoma and Texas; the Western region of the United States includes thirteen states: Montana, Wyoming, Colorado, New Mexico, Idaho, Utah, Arizona, Nevada, Washington, Oregon California, Alaska and Hawaii.
